# Religious Identity and its Relation to Health-Related Quality of Life and COVID-Related Stress of Refugee Children and Adolescents in Germany

**DOI:** 10.1007/s10943-023-01966-6

**Published:** 2023-12-15

**Authors:** P. Schmees, J. Braig, Y. Kilinc, H. Nilles, U. EL-Awad, D. Kerkhoff, Z. Demir, J.-E. Rueth, A. Lohaus, H. Eschenbeck

**Affiliations:** 1 Department of Educational Psychology and Health Psychology, University of Education Schwäbisch Gmünd, Schwäbisch Gmünd, Germany; 2 Department of Psychology, Bielefeld University, Bielefeld, Germany; 3 Clinical Psychology and Psychotherapy, Medical School Berlin, Berlin, Germany; 4 Institute for Interdisciplinary Research on Conflict and Violence, Bielefeld University, Bielefeld, Germany

**Keywords:** Religious identity, Well-being, Quality of life, COVID-19, Refugee children and adolescents

## Abstract

Research shows that religious identity is associated with health. The aim of this study was to understand the role of religious identity for refugee minors’ health in greater detail. Middle Eastern refugee minors resettled in Germany and aged 8–18 years completed questionnaires at baseline (T1, *n* = 246) and follow-up (T2, *n* = 122) measurement between 2019 and 2022. Religious identity was assessed with a 4-item measure (Cronbach’s *α* = .89). Associations of religious identity at T1 with health-related quality of life (HRQoL) at T1, change in HRQoL from T1 to T2, and perceived COVID-related stress at T2, as well as the mediating role of resources were examined. The results showed a positive association between religious identity and HRQoL, which was partially mediated by integration into peer group, but not by ethnic identity, sense of coherence or religious practice. No significant associations between religious identity and change in HRQoL or COVID-related stress occurred. Therefore, cross-sectional analyses support the beneficial role of religious identity for HRQoL and the crucial mediating role of integration into peer group, suggesting the promotion of religious identity or peer group integration. However, the absence of significant effects on change in HRQoL from T1 to T2 and COVID-related stress at T2 do not allow drawing any long-term conclusions.

**Supplementary Information:**

The online version contains supplementary material available at 10.1007/s10943-023-01966-6.

## Introduction

Refugee children and adolescents possess many skills and strengths, partly due to their cultural background and their flight experience (Marley & Mauki, 2019). Further, they are exposed to many challenges (e.g., Gavranidou et al., 2008; UNICEF, 2017). To cope with these challenges, resources are of particular importance. In contrast to a predominantly deficit-oriented approach that focuses on reported stresses and health impairments (The Federal Government Commissioner for Migration, Refugees and Integration, 2019; Jafari et al., 2022), this study takes a positive and resource-oriented perspective and explores the empowering and sustainable potential of religious identity. Religious identity describes the extent to which self-perception is associated with religion (Zagumny, 2013). The extent of religious identity is positively related to health-related quality of life (HRQoL) and is more salient for refugee children than for native children in Germany (Schmees et al., 2022). However, little is known about whether religious identity affects changes in HRQoL and if it can also help refugee children and adolescents in Germany to experience less COVID-related stress. Moreover, the underlying mechanisms that may explain the association between religious identity and HRQoL have not been conclusively investigated.

### Religiosity as a Resource

Religion plays an important part in human history and in society. Nevertheless, no consensus on the definition of religiosity exits in the field of research (Zinnbauer & Pargament, 2005). In the present study religiosity is understood as a personal shaping and life practice of religion. Religion comprises religious affiliation and a community that shares belief norms, traditions, rituals, and texts (Utsch et al., 2017). Religiosity as a resource can be helpful in coping with challenging events, both for adults (de Diego-Cordero et al.,^ 2022^; Emery et al., 2022; Schweitzer et al., 2007) and adolescents (Chow et al., 2021; EL-Awad et al., 2022), depending on cultural background (Tan et al., 2022). Religiosity or religious coping is related to various health outcomes, that is higher life satisfaction and quality of life (Adam & Ward, 2016; Tan et al., 2022), as well as less perceived stress and depression (Ozeto et al., 2021; Ronneberg et al., 2016). The positive association between religiosity and health can be explained by various underlying mechanisms, for example, social capital (Shapiro, 2022) or meaning-making (Krok et al., 2021), which are described in more detail below. Although religiosity can be a significant resource for refugees, it is under-researched and receives little attention in current scientific discourse (Scharpf et al., 2021). Furthermore, findings on religious beliefs as a resource are inconsistent and the underlying mechanisms are complex (Fazel et al., 2012). One possible explanation for the inconsistent findings is that whether religiosity can be experienced as a resource depends on the context or environmental conditions and society (Sabatier et al., 2011; Torralba, 2021). Religion can also lead to separation, if a religious affiliation makes integration into society more difficult, which in turn can have negative effects on development (Phalet et al., 2018). Religious affiliations can also lead to conflicts and tensions with people of other religious affiliations, for example, in accommodations for refugees (Powroznik, 2021). This raises the question of whether religiosity can be a resource for refugee children and adolescents in the environmental conditions and the society in Germany.

### Religion in Germany

The German state is neutral and open to all religious and ideological beliefs (Federal Constitutional Court, 2020). However, Germany’s basic law does not provide for a strict separation between state and religion, which is why the state cooperates with religious communities, especially Christian denominations, for example, to organize religious education in state schools (Körs et al., 2022). Despite declining numbers of religiously affiliated people, about half of people with German citizenship belong to a Christian church (Research group on worldviews in Germany, 2021). Islam forms the third largest faith community in Germany after Christian denominations, accounting for approximately 3% to 6% of the total population (Federal Government Expert Commission on the Framework Conditions for Integration Potential, 2021; Research group on worldviews in Germany, 2021). In contrast to the resource-oriented perspective of this article, Islam is often portrayed in public debates in Germany as threatening, deficient, and in contradiction with Christian culture (Federal Government Expert Commission on the Framework Conditions for Integration Potential, 2021).

### Religiosity of Refugee Children and Adolescents in Germany

In 2020, about 510,000 refugee children and adolescents seeking asylum lived in Germany and the main countries of origin were Syria, Iraq, and Afghanistan (Federal Statistical Office, 2021). A previous report using data from the YOURHEALTH project in Germany (on which the present manuscript is also based), showed that refugee children and adolescents from the Middle East overall have fewer social and personal resources available than non-refugee children and adolescents (Schmees et al., 2022). The resource religious identity contrasted with this observation, as refugee children and adolescents identified more strongly with their religion than non-refugee children and adolescents, which may be due to the cultural circumstances in their countries of origin. Accordingly, Fleischmann (2022) showed that Muslim minorities in European societies differ from non-Muslim people in the strength and stability of religiosity or religious identity as well as practices and attitudes. In addition to a stronger religious identity of refugee children and adolescents, a pronounced positive association between religious identity and HRQoL was found (Schmees et al., 2022). Furthermore, EL-Awad et al. (2022) showed that stronger religiosity among refugee and immigrant youth from the Middle East in Germany was associated with lower levels of internalizing symptoms and that refugee youth’s religious activity buffers the positive association between traumatic events and internalizing symptoms. To extend the findings, this study examines whether religious identity is not only cross-sectionally associated with HRQoL but is also related to positive change in HRQoL over time.

### Mechanisms by Which Religiosity Affects Well-Being and Health

Due to the beneficial role of religiosity, it is discussed whether it should be included in health promotion (Kizilhan, 2015; Shapiro, 2022; Surzykiewicz & Maier, 2020; Utsch, 2015). To use religiosity in health promotion, it is necessary to identify the underlying mechanisms explaining its beneficial role. Two popular mediating mechanisms to be highlighted are the social capital and the meaning-making system.

#### Integration Into Peer Group as a Mediator

The mediating effect of social capital, which implies social resources, is explained by the fact that religiosity can have a unifying effect, bringing people together through religious communities and finding social structures and mutual social support in these communities, which in turn is related to positive health (Shapiro, 2022). Previous studies already investigated this mechanism. For example, a study with African American adults in the USA showed the mediating role of belonging and tangible support in the association between religious behavior and physical functioning or depression (Holt et al., 2014). In the present study, the mediating influence of peer group integration, as an important component of social capital, is examined.

#### Sense of Coherence as a Mediator

The mediating effect of meaning-making can be explained by the fact that people can gain a sense of purpose, goals and answers to existential questions through religiosity, and this in turn can be associated with a greater well-being (Zarzycha et al., 2020). For example, using a sample of youths in Poland, it was shown that meaning-making mediates the relationship between religiosity and life satisfaction as well as positive and negative affect (Krok et al., 2021). Especially after stressful life events or situations which violate global meaning, such as an escape or a pandemic, meaning-making can help to restore global life meaning, for example by changing the perspective on the situation, transforming the meaning of events or reforming one’s beliefs (Park et al., 2013). Together with comprehensibility and manageability, meaningfulness represents the salutogeneic construct sense of coherence, which describes how humans are able to cope with stressors and which is associated with positive health (Antonovsky, 1979; Carlén et al., 2020). Therefore, analogous to previous studies (Cowlishaw et al., 2013; Krok et al., 2016), sense of coherence is considered as a mediator in this study.

#### Ethnic Identity and Religious Practice as Mediators

In addition, possible explanatory effects of ethnic identity and religious practice are considered in the present study. It is assumed that children and adolescents who identify more strongly with their religion also identify more strongly with their culture of origin (as religion is usually more prevalent in the included countries of origin), which in turn may explain the beneficial role of religiosity, because ethnic identity can be a protective factor for refugee children (Marley & Mauki, 2019). In a sample of adult Middle Eastern migrants in Australia, Hashemi et al. (2020) already showed, among other things, an indirect effect of religious identity on psychological well-being through social connectedness with ethnic community as well as through social support. Further, it is assumed that children and adolescents who identify more with their religion, practice their religion more frequently (e.g., by praying, by attending religious services) and that this results in a better HRQoL, as previous studies showed a beneficial role of religious practice for health outcomes (e.g., Michaels et al., 2022). In the present study, it is assumed that religious practice is positively associated with religious identity although not all minors who identify with their religion also practice their religion (Leszczensky & Pink, 2020).

### Religiosity as a Resource During the COVID-19 Pandemic

Moreover, religiosity can serve as a resource in times of crisis and can be helpful in coping with challenges (e.g., support obtained from religious and spiritual beliefs or activities can help children coping with the loss of a parent, Greeff & Human, 2004). A major global crisis was the COVID-19 pandemic which has led to new challenges (e.g., fear of infection, uncertainty, isolation, and restrictions). Children and adolescents are particularly affected by this crisis, as it has a strong impact on their daily lives and affects social contacts and schooling (UNESCO Institute for statistics, 2021). A population-based study showed that the HRQoL of children and adolescents in Germany increasingly deteriorated during the pandemic (Ravens-Sieberer et al., 2021). In contrast, the life satisfaction of refugees improved in the early period of the pandemic (Niehues, 2022). Among other factors, one possible explanation for this contradictory finding may be religiosity, a resilience resource in times of crisis that is more available to refugee children and adolescents from the Middle East (Brown & Floyd, 2022; Schmees et al., 2022). Religiosity can be helpful in the COVID-19 pandemic by reducing stress, anxiety, and depressive symptoms as well as strengthening resilience and hope (de Diego-Cordero et al., 2022; Pirutinsky et al., 2020; Schnabel & Schieman, 2022). From this consideration, it is hypothesized that refugee children and adolescents who identify more strongly with their religion experience less COVID-related stress.

### The Present Study

This study investigates whether religious identity is a short- and long-term resource for refugee children and adolescents in Germany and whether mediating variables can explain potential associations with health. By understanding the relationship between religious identity and health, this study aims to provide perspectives for target group specific health promotion. The following assumptions are tested:

1. Religious identity is positively associated with (1.1) HRQoL and (1.2) change in HRQoL and negatively associated with (1.3) perceived COVID-related stress at follow-up measurement.
2. Ethnic identity, sense of coherence, integration into peer group, or religious practice partially mediate the association between religious identity and (2.1) HRQoL as well as (2.2) change in HRQoL.

## Method

Data were collected as part of the YOURGROWTH study which is part of the YOURHEALTH (YOUng Refugee HEALTH) collaborating project. Ethical approval for the project was obtained from the ethics committees of the participating universities. Informed consent was obtained from both parents and minors prior to the study in written form. Inclusion criteria were that the children and adolescents were between 8 and 18 years old at the time of baseline measurement and reported Syria, Iraq, or Afghanistan as their country of origin.

### Procedure

The YOURGROWTH study is a longitudinal questionnaire study, of which data from two survey waves were analyzed for this article. Surveys were conducted in three regions in Germany (Baden-Wuerttemberg, North Rhine-Westphalia, Hamburg). The first contact with the children and adolescents was made with the help of employees of interested schools, accommodations for refugees, or sports and youth clubs. At baseline measurement, which took place partly before and partly during the pandemic (T1; 2019–2020), children and adolescents completed the questionnaires either on tablets or in a paper–pencil version in a group setting in schools, in accommodations for refugees, or in sports and youth clubs. Participants had the options of choosing the language of the questionnaire (German, Arabic, Kurmandci, Sorani, Dari, or Pashto), listening to the questions and answers via an audio button or getting help from trained stuff (preferably with knowledge in their first language). The follow-up measurement (T2; 2020–2022), which attempted to reconnect with all participants, was conducted during the pandemic and on average 15 months (*M* = 15.17, *SD* = 5.11) after the baseline measurement. At follow-up, children and adolescents who could be contacted again answered the questions at home either online or as a paper–pencil questionnaire. When answering the questions, participants were generally in contact via phone with trained staff (preferably with knowledge in their first language). After each measurement, participants received contact information of institutions they can turn to for mental health support.

### Participants

Altogether, 554 children and adolescents participated at the baseline measurement and 268 children and adolescents participated at the follow-up measurement. Data from *n* = 308 minors at baseline measurement were excluded from the analyses because of inaccurate demographic information (*n* = 140) or missing information on the scales to assess religious identity, HRQoL, ethnic identity, sense of coherence, integration into peer group, or religious practice (*n* = 168). Data from *n* = 146 minors at follow-up measurement were excluded from the analyses because of missing information at baseline survey (*n* = 138) or on the scale to assess HRQoL at T2 (*n* = 8). This resulted in a sample of *N* = 246 children and adolescents at baseline and *N* = 122 children and adolescents at follow-up measurement.

#### Participants’ Characteristics

The sociodemographic data of the participants included in both the baseline survey and the follow-up survey did not differ significantly from the data of the participants included only in the baseline survey. Detailed information on participants’ characteristics is shown in Table 1. All participants arrived in Germany between 2014 and 2020, but most arrived between 2015 and 2016 (T1: *n* = 96, T2: *n* = 50). However, since many participants had difficulty providing precise information about their time of arrival in Germany, this information should be treated with caution and is not considered further in the analyses.

**Table 1:** Participant characteristics at baseline and follow-up measurement

Variable	Baseline *N* = 246	Follow-up *N* = 122
Age (in years), *M* (*SD*)	12.83 (2.80)	14.00 (2.81)
*Gender, n (%)*		
Male	139 (56)	65 (53)
Female	107 (44)	56 (46)
Missing	–	1 (1)
*Country of origin, n (%)*		
Syria	152 (62)	81 (66)
Iraq	65 (26)	23 (19)
Afghanistan	29 (12)	18 (15)
*Religious affiliation, n (%)*		
Islam	193 (78)	100 (82)
Yazidism	45 (18)	19 (16)
Other	6 (2)	33 (1)
Unclear	2 (2)	–
*Unaccompanied, n (%)*	5 (2)	1 (1)
Missing	16 (6)	9 (7)
*Survey location, n (%)*		
Baden-Wuerttemberg	89 (36)	59 (48)
North Rhine-Westphalia	118 (48)	30 (25)
Hamburg	39 (16)	33 (27)

### Measures

Questionnaires that were not available in the required languages were translated into German, Arabic, Kurmandci, Sorani, Dari, and Pashto in a back-and-forth translation process, which was basically in line with the steps for scale translation outlined in Koenig and Al Zaben (2021). Most children chose the German version of the self-report questionnaire (T1: 78%; T2: 72%). All questionnaires, except the scale to assess COVID-related stress, were assessed at both survey waves. Internal consistencies (Cronbach’s α) for each scale are reported below specifically for the relevant survey waves.

#### Religious Identity

Religious identity was assessed with 4 items (“My religion is an important part of myself”, “It bothers me when someone speaks ill of my religion”, “My religion is dear to me”, “I feel like I am part of my religion”) on a 5-point scale (Fleischmann et al., 2019). Internal consistency was excellent for the sample at T1 (*α* = 0.89).

#### Health-Related Quality of Life

HRQoL was assessed using the KIDSCREEN-10 (The KIDSCREEN Group Europe, 2006). It consists of 10 items (e.g., “Please think about last week. … Have you been full of energy?”), using a 5-point response format. Internal consistencies were acceptable for the sample at T1 (*α* = 0.78) and T2 (*α* = 0.78).

#### Perceived COVID-Related Stress

Perceived COVID-related stress was assessed with the *Perceived COVID-Related Stress Scale for Children and Adolescents* that asks about stress related to the coronavirus itself, as well as stress related to COVID-19 limitations. It comprises four items (e.g., “I feel bad when I think about the coronavirus or hear information about it”, “I feel lonely because of the Corona time”), which were adapted from the YuCo-Study (Andresen et al., 2020). A 5-point response format was used. Internal consistency was comparatively low for the sample at T2 (*α* = 0.59).

#### Ethnic Identity

Ethnic identity was assessed using the *Ethnic and National Identity Questionnaire for Children and Adolescents* (Leszczensky & Gräbs Santiago, 2014). It consists of 4 items measuring the emotional attachment to the country of origin (e.g., “I feel that I am a part of my country of origin”) with a 5-point response format. The items to assess ethnic and religious identity were formulated in a comparable manner. Internal consistency was acceptable for the sample at T1 (*α* = 0.78).

#### Sense of Coherence and Integration into Peer Group

Sense of coherence and integration into peer group were assessed using subscales of the *Questionnaire to Assess Resources for Children and Adolescents* (QARCA; Lohaus & Nussbeck, 2016). Each subscale comprises 6 items that are answered on a 4-point scale. Internal consistencies of the subscales sense of coherence (*α* = 0.80) and integration into peer group (*α* = 0.80) were good for the sample at T1.

#### Religious Practice

Religious practice was measured by 2 items (“How often do you pray?”, “How often do you visit a place of worship (e.g., a church, mosque, or temple)?”), using a 5-point scale. The 2 items had a moderate positive inter-item-correlation for the sample at T1 (*r* = 0.49).

### Data Analyses

Mean values were calculated for complete cases for all scales or subscales, using IBM SPSS Version 27. In case of less than 20% missing values of one scale or subscale, missing values were replaced by the minor´s mean value. A change score was calculated to measure the change in HRQoL (HRQoL T2–HRQoL T1), as it is the most direct measure of change over time, that is easy to interpret (van Buuren, 2018). Bivariate Spearman correlations were calculated to test Hypothesis 1. G*Power 3.1. analyses indicate that sample sizes (*N* = 246, *N* = 122, *N* = 111) offered sufficient power (> 0.80) to detect a medium-sized effect with an alpha level of *p* = 0.01 (Faul et al., 2009).

#### Mediation Analyses

Mediation analyses (Hypothesis 2) were performed using the SPSS PROCESS Macro (Hayes, 2018). Mediation analyses (model 4) with one mediator each were performed with bootstrap procedures (10,000 bootstrap resamples). To prevent false-positive results due to multiple testing, a confidence interval of 99% was set in accordance with the Bonferroni correction. Monte Carlo power analyses (Schoemann et al., 2017) for mediation analyses with one mediator (*r* = 0.30, *α* = 0.01) indicated that our final baseline sample (*N* = 246) was well powered to detect indirect effects in cross-sectional design (power = 0.85), but our final follow-up-sample (*N* = 122) was not (power = 0.39). Therefore, we do not interpret non-significant results on the indirect effects in the longitudinal design. The relationship of all variables involved in the mediation analysis was approximately linear, as assessed by visual inspection of the scatterplots. In the cross-sectional design, we also conducted a parallel mediation analysis in which all four mediators were included in the model simultaneously. In the longitudinal design, this procedure was not feasible due to the smaller sample and thus lower power.

#### Covariates

Age (Chan et al., 2015; King et al., 2020), religious affiliation (Phalet et al., 2018), gender (Le et al., 2007), and time interval between T1 and T2 may be associated with religiosity, HRQoL, change in HRQoL, and COVID-related stress. Furthermore, due to different regulations and conditions in the federal states, the survey location (Baden-Wuerttemberg, North Rhine-Westphalia, Hamburg) may also be associated with the variables studied. Preliminary analyses revealed no meaningful associations of gender and age with the outcome variables. Also, no significant correlation between change in HRQoL and the time interval between the two measurements occurred (*r* = − 0.04, *p* = 0.685). However, religious affiliation and the survey location showed significant associations with relevant variables (*p* < 0.001) and were consequently included as covariates in mediation analyses. As most participants stated Islam as their religious affiliation (see Table 1), religious affiliation was included as dichotomized variable (1 = Islam, 0 = other). The survey location was included as dummy coded variables.

## Results

### Descriptive Statistics and Correlations (Hypothesis 1)

Descriptive statistics and correlations among studied variables are displayed in Table 2. Correlations revealed positive associations between religious identity and HRQoL (T1) and no associations between religious identity and change in HRQoL as well as between religious identity and COVID-related stress. Accounting for the time distance between measurements as a moderator (*R*^*2*^ = 0.03, *F*(3, 90) = 0.83, *p* = 0.479) did not change the lack of association between religious identity and COVID-related stress. Thus, Hypothesis 1.1 is confirmed, but Hypotheses 1.2 and 1.3 are not confirmed.

**Table 2:** Means, standard deviations, and correlations

Variable	*n*	*M*	*SD*	1	2	3	4	5	6	7	8
1. Religious identity (T1)	246	4.33	0.98								
2. HRQoL (T1)	246	3.62	0.73	0.25**							
3. HRQoL (T2)	122	3.88	0.66	0.15	0.33**						
4. Change in HRQoL (T2-T1)	122	0.26	0.83	0.01	−0.59**	0.50**					
5. COVID-related stress (T2)	111	3.14	1.04	0.01	−0.04	−0.18	−0.10				
6. Ethnic identity (T1)	246	4.11	0.96	0.44**	0.21**	0.12	−0.07	0.16			
7. Sense of coherence (T1)	246	2.70	0.67	0.20**	0.44**	0.12	−0.30**	−0.03	0.18**		
8. Integration into peer group (T1)	246	2.89	0.68	0.23**	0.50**	0.17	−0.23**	−0.09	0.17**	0.51**	
9. Religious practice (T1)	246	2.65	1.39	0.30**	−0.05	−0.12	0.04	0.00	0.16*	0.10	0.05

*p < .05, **p < .001

Positive associations were found between religious identity and all possible mediators as well as between HRQoL (T1) and all possible mediators except religious practice. Regarding change in HRQoL, negative associations were found with sense of coherence and integration into peer group. No associations were found between COVID-related stress and any variable.

### Mediation Models (Hypothesis 2)

Mediation analyses were conducted to examine whether the associations between religious identity at T1 and HRQoL at T1 (Hypothesis 2.1) and between religious identity at T1 and change in HRQoL from T1 to T2 (Hypothesis 2.2) were mediated by ethnic identity, sense of coherence, integration into peer group, and religious practice.

Results of simple mediation analyses indicate that the positive relationship between religious identity and HRQoL (controlling for religious affiliation and the survey location) was partially mediated by integration into peer group but not by ethnic identity, sense of coherence, and religious practice (see Table 3). A parallel mediation analysis with all mediators in one model, showed comparable results. The indirect effect through integration into peer group was significant, but all other indirect effects were non-significant (see ESM1). Thus, Hypothesis 2.1 was partly confirmed.

**Table 3:** Standardized path coefficients for total, direct, and indirect effects of simple mediation analyses with religious identity as independent variable and HRQoL as dependent variable

Mediator	Path a	Path b	Path c’ (direct effect)	Path c (total effect)	Path a x b (indirect effect)
Ethnic identity	0.48 (*p* < .001)	0.12 (*p* = .081)	0.19 (*p* = .007)	0.25 (*p* < .001)	0.06 Cl [− .0360; .1850]
Sense of coherence	0.16 (*p* = .010)	0.34 (*p* < .001)	0.19 (*p* = .001)	0.25 (*p* < .001)	0.05 Cl [− .0139; .1371]
Integration into peer group	0.27 (*p* < .001)	0.39 (*p* < .001)	0.16 (*p* = .014)	0.25 (*p* < .001)	0.10 Cl [.0214; .2039]
Religious practice	0.31 (*p* < .001)	− 0.14 (*p* = .025)	0.29 (*p* < .001)	0.25 (*p* < .001)	− 0.04 Cl [− .1046; .0037]

*N* = 246, Covariates: Religious affiliation and survey location

Simple mediation analyses with change in HRQoL as dependent variable showed no total effect between religious identity and change in HRQoL and no indirect effects (see Table 4). Therefore, Hypothesis 2.2 was not confirmed.

**Table 4:** Standardized path coefficients for total, direct, and indirect effects of simple mediation analyses with religious identity as independent variable and change in HRQoL as dependent variable

Mediator	Path a	Path b	Path c’ (direct effect)	Path c (total effect)	Path a x b (indirect effect)
Ethnic identity	0.46 (*p* < .001)	− 0.06 (*p* = .584)	− 0.03 (*p* = .795)	− 0.06 (*p* = .563)	− 0.03 Cl [− .1868; .1271]
Sense of coherence	0.07 (*p* = .468)	− 0.25 (*p* = .008)	− 0.04 (*p* = .680)	− 0.06 (*p* = .563)	− 0.02 Cl [− .1276; .0666]
Integration into peer group	0.16 (*p* = .088)	− 0.19 (*p* = .036)	− 0.02 (*p* = .805)	− 0.06 (*p* = .563)	− 0.03 Cl [− .1573; .0289]
Religious practice	0.35 (*p* < .001)	0.07 (*p* = .506)	− 0.08 (*p* = .440)	− 0.06 (*p* = .563)	0.02 Cl [− .0703; .1217]

*N* = 122, Covariates: religious affiliation and survey location

## Discussion

The aim of the present study was to understand the association between religious identity and health-related quality of life and COVID-related stress of refugee children and adolescents in Germany in more detail. More specifically, we aimed to investigate which variables may mediate a possible association between religious identity and HRQoL as well as between religious identity and change in HRQoL over time.

### The Relation of Religious Identity to HRQoL

In cross-sectional analyses, as hypothesized, religious identity was positively associated with HRQoL (T1) of refugee minors in Germany (Hypothesis 1.1). In line with previous research (e.g., Schmees et al., 2022; Tan et al., 2022), we can conclude that refugee minors in Germany, who identify more strongly with their religion, experience better HRQoL. This association was partially mediated by integration into peer group, but not by ethnic identity, sense of coherence, and religious practice (Hypothesis 2.1). In the following sections, we discuss the possible underlying mechanisms that may account for the results with respect to each mediator considered.

#### The Mediating Effect of Integration into Peer Group

Analogous to studies with samples from other age groups and from different countries (Hashemi et al., 2020; Holt et al., 2014), refugee minors in Germany, who identified more strongly with their religion, were better integrated into a peer group, which was associated with better HRQoL. The mediating effect of integration into peer group confirms previous studies and the theoretical assumption that social capital can explain the positive association between religiosity and positive health outcomes (Shapiro et al., 2022; Ysseldyk et al., 2013). The findings suggest that social capital is a key explanatory factor for the association of religiosity with health outcomes, rather than the meaning-making system, ethnic identity, or religious practice.

#### No Mediating Effects of Ethnic Identity and Sense of Coherence

Minors who identified more strongly with their religion also identified more strongly with their country of origin and reported a higher sense of coherence, but this showed not to be related to a better HRQoL. The correlation found between HRQoL and ethnic identity and between HRQoL and sense of coherence does not seem to be notable in the mediation analysis. One possible explanation for these results can be, that religious identity implicates the factors, for example sense of belonging or sense of purpose, which explain the positive correlation between ethnic identity and HRQoL as well as sense of coherence and HRQoL. This presumption should be investigated in future studies.

#### No Mediating Effect of Religious Practice

Further, no mediating effect of religious practice was found. Religious practice was related to religious identity but not to HRQoL, which is inconsistent with previous studies showing a positive association between religious practice and health outcomes (e.g., Michaels et al., 2022). The absence of a correlation between religious practice and HRQoL in this study can be explained by the circumstance that the culture in Germany is shaped by Christianity. Religious holidays in Germany are based only on Christianity and most places of worship in Germany are Christian; places of worship of other denominations are rare (Federal Government Expert Commission on the Framework Conditions for Integration Potential, 2021). Actively practicing a religion other than Christianity by visiting houses of worship and celebrating religious holidays of other denominations, is accordingly more difficult. On top of that, according to a large-scale study, one-fifth of Germans have negative attitudes toward Muslims (Zick & Küpper, 2021). It is possible that minors who practice their religion publicly in Germany experience more exclusion and discrimination and that this makes praying in everyday life (e.g., praying several times a day in school) more difficult (Vang et al., 2019). Making it more difficult to practice religion in Germany may explain why no association between religious practice and HRQoL was found in this study.

### The Relation of Religious Identity to Change in HRQoL and COVID-Related Stress

Regarding longitudinal analyses, contrary to Hypotheses 1.2 and 2.2, no association between religious identity and change in HRQoL, and no mediating effect was found. No association was shown between religious identity and HRQoL at T2, either. Accordingly, the results suggest that there is a cross-sectional positive association between religious identity and HRQoL of refugee children and adolescents in Germany, but that religious identity does not have a significant long-term positive effect on changes in HRQoL over time. In addition, no association between religious identity and COVID-related stress was found (Hypothesis 1.3). Thus, the results suggest that refugee children and adolescents who identify more strongly with their religion do not experience less COVID-related stress one year later than children and adolescents who identify less with their religion. Accordingly, religious identity does not appear to serve as a long-term resilience resource in the COVID-19 pandemic.

#### Possible Explanations for the Absence of Long-Term Effects

One explanation for the lack of positive longitudinal associations is that religious identity develops steadily across the lifespan (Zagumny, 2013). Particularly during childhood and adolescence identity can change rapidly, as identity development is one of the most important developmental tasks during adolescence (Branje et al., 2021). Further, religious cognitions can change rapidly (e.g., the change of concepts of God and soul), due to cognitive development in childhood (Boyatzis, et al., 2013). Moreover, in addition to identity development in childhood and adolescence, certain characteristics of the specific sample of refugee children and adolescents as well as the particular survey period affected by the pandemic may explain the absence of long-term effects on change in HRQoL over time and COVID-related stress.

#### Changing Life Circumstances and Challenges of Refugee Children and Adolescents

Children and adolescents are generally confronted with many changes and challenges (e.g., mental and somatic changes during puberty or developmental tasks such as identity development). Refugee children and adolescents face additional challenges and often must adapt to changing life circumstances, as these are often related to asylum decisions or length of stay and can change quickly. The housing situation (Baier & Siegert, 2018), health care (Lindner, 2022) as well as regulations on compulsory schooling and access to the education system (de Paiva Lareiro, 2019) vary between places of residence, residence status, and length of stay. Correspondingly, Zettl et al. (2021) showed that refugee adults experience more identity diffusion compared to first- and second-generation migrants in Germany. These challenges and potential changes may also result in more profound changes in religious identity of refugee children and adolescents.

#### Changing Life Circumstances and Challenges During the COVID-19 Pandemic

In addition, the COVID-19 pandemic, which caused additional changes in living conditions and other challenges, started in March 2020, so that the baseline survey took place partly before and partly during the pandemic. The follow-up survey took place throughout the COVID-19 pandemic. In Germany, the COVID-19 pandemic in 2020 and 2021 was accompanied by an alternation between classroom-based and restricted classroom-based education in schools, which varied by federal state and school. In 2020, there were overarching contact restrictions (e.g., only a maximum of two households were allowed to meet, leisure activities were restricted, social distancing rules were introduced), which were intensified depending on local incidence rates (Cheng et al., 2020). In 2021, contact restrictions were predominantly based on local incidence rates. Thus, in the wake of the pandemic and the refugee status, both associated with challenges and changes, other factors may have had an additional impact on the change in HRQoL and COVID-related stress beyond religious identity. For example, changing life circumstances (e.g., housing situation, health care, schooling) due to asylum decisions or length of stay, housing situations or changing schooling restrictions and contact restrictions during the pandemic may have an impact on the change in HRQoL and COVID-related stress. Future studies could examine which other variables, life circumstances or religious coping strategies are significant for the change in HRQoL and COVID-related stress.

### Implications for Future Research and Practice

To our knowledge, this is the first study to examine religious identity among refugee children and adolescents in Germany in more detail. A representative survey showed that religious identity is important for the identity of people in Germany and Switzerland and has the potential to both connect and exclude people (Liedhegener et al., 2019). Especially for refugee children and adolescents, religious identity is an important resource, yet it receives little attention in research and practice (Schmees et al., 2022).

#### Religiosity and Integration into Peer Group in Health Promotion

For practice, it is important to provide answers to what extent people working in the health and social system can integrate religious identity as a resource in their work with refugee children and adolescents. Cross-sectional analyses indicate that religious identity and integration into peer group are health resources for refugee children and adolescents. Accordingly, a cost-effective approach to health promotion can be working with religious identity or the involvement of religious communities, but it can also directly address the promotion of integration into peer group (Klein & Albani, 2007; Shapiro et al., 2022). However, since no significant effects on change in HRQoL were found, religious identity and integration into peer group do not lead to an improvement in HRQoL in the long term, but rather represent indicators of HRQoL. This study is a beginning to explore the role of religiosity for the health of refugee children and adolescents. Further research is needed to examine the long-term impact of religious identity on health of refugee children and adolescents. Also, possible variables that are relevant for the strength of the association between religious identity and HRQoL should be identified, and the potentially crucial influence of contextual and living conditions in this regard should be explored.

### Study Limitations

One of the most important limitations of this study is that only two survey waves were available for the analysis. As a result, long-term developmental trajectories remain unclear. Further, the power differed markedly between the cross-sectional and longitudinal sample, with a critical power for longitudinal mediation analyses. Thus, only significant results of the longitudinal mediation analyses could have been interpreted, whereas the non-significant results cannot be interpreted. In addition, besides the change in HRQoL between measurement waves, the change in other variables (e.g., religious identity) was not considered, because of a small sample size. Due to limited data quality, length of stay could not be included as an influencing factor. In addition, the internal reliability of the scale to assess perceived COVID-related stress is insufficient, which is why the results on the association between religious identity and COVID-related stress must be interpreted with caution.

## Conclusion

Religious identity was positively associated with HRQoL of refugee minors in Germany. This association was partially mediated by integration into peer group, but not by ethnic identity, sense of coherence, and religious practice. Therefore, religious identity and integration into peer group can be considered as factors to address when promoting health-related quality of life among refugee children and adolescents. No significant effects of religious identity on change in HRQoL and COVID-related stress were found. We assume that other factors (e.g., identity development, living conditions, COVID-situation) play a relevant role for the change in HRQoL, COVID-related stress and the association between religious identity and change in HRQoL of refugee children and adolescents, which should be investigated in future studies.

### Supplementary Information

Below is the link to the electronic supplementary material.

Supplementary file1 (DOCX 13 kb)

## References

[CR1] Adam, Z., & Ward, C. (2016). Stress, religious coping and wellbeing in acculturating Muslims. *Journal of Muslim Mental Health, 10*(2). 10.3998/jmmh.10381607.0010.201

[CR2] Andresen, S., Lips, A., Möller, R., Rusack, T., Schröer, W., Thomas, S., & Wilmes, J. (2020). *Erfahrungen und Perspektiven von jungen Menschen während der Corona-Maßnahmen. Erste Ergebnisse der bundesweiten Studie JuCo [Experiences and perspectives of young people during Corona measures. First results of the nationwide study JuCo].* Universitätsverlag Hildesheim. 10.18442/120

[CR3] Antonovsky A (1979). Health, stress and coping.

[CR4] Baier, A., & Siegert, M. (2018). *Die Wohnsituation Geflüchteter. Ausgabe 02|2018 der Kurzanalysen des Forschungszentrums Migration, Integration und Asyl des Bundesamtes für Migration und Flüchtlinge [The housing situation of refugees. Issue 02|2018 of the brief analyses of the research center migration, integration and asylum of the federal office for migration and refugees].* https://www.bamf.de/SharedDocs/Anlagen/DE/Forschung/Kurzanalysen/kurzanalyse11_iab-bamf-soep-befragung-gefluechtete-wohnsituation.pdf?__blob=publicationFile&v=12

[CR5] Boyatzis CJ, Paloutzian RF, Park CL (2013). Religious and spiritual development in childhood. Handbook of the Psychology of Religion and Spirituality.

[CR6] Branje S, de Moor EL, Spitzer J, Becht AI (2021). Dynamics of identity development in adolescence: A decade in review. Journal of Research on Adolescence.

[CR8] Brown SA, Floyd FJ (2022). Subjective religiosity as resilience to stressful life events in middle-aged and older African Americans. Journal of Religion, Spirituality and Aging.

[CR9] Carlén K, Suominen S, Lindmark U, Saarinen MM, Aromaa M, Rautava P, Sillanpää M (2020). Sense of coherence predicts adolescent mental health. Journal of Affective Disorders.

[CR10] Chan M, Tsai KM, Fuligni AJ (2015). Changes in religiosity across the transition to young adulthood. Journal of Youth and Adolescence.

[CR11] Cheng, C., Barceló, J., Hartnett, A., Kubinec, R., & Messerschmidt, L. (2020). *COVID-19 government response event dataset (CoronaNet v1.0)*. https://www.coronanet-project.org 10.1038/s41562-020-0909-7 32576982

[CR12] Chow MISP, Hashim AH, Guan NC (2021). Resilience in adolescent refugees living in Malaysia: The association with religiosity and religious coping. International Journal of Social Psychiatry.

[CR13] Cowlishaw S, Niele S, Teshuva K, Browning C, Kendig H (2013). Older adults' spirituality and life satisfaction: A longitudinal test of social support and sense of coherence as mediating mechanisms. Ageing and Society.

[CR14] de Diego-Cordero R, de, Ávila-Mantilla, A., Vega-Escaño, J., Lucchetti, G., & Badanta, B.  (2022). The role of spirituality and religiosity in healthcare during the COVID-19 pandemic: An integrative review of the scientific literature. Journal of Religion and Health.

[CR15] de Paiva Lareiro, C. (2019). *Ankommen im Deutschen Bildungssystem - Bildungsbeteiligung von geflüchteten Kindern und Jugendlichen. Ausgabe 02|2019 der Kurzanalysen des Forschungszentrums Migration, Integration und Asyl des Bundesamtes für Migration und Flüchtlinge [Arriving in the German education system - Educational participation of refugee children and adolescents. Issue 02|2019 of the brief analyses of the research center migration, integration and asylum of the federal office for migration and refugees].* https://www.bamf.de/SharedDocs/Anlagen/DE/Forschung/Kurzanalysen/kurzanalyse2-2019-ankommen-im-deutschen-bildungssystem.pdf?__blob=publicationFile&v=12

[CR16] EL-Awad, U., Fathi, A., Lohaus, A., Petermann, F., & Reinelt, T.  (2022). Different relations of religion and mental health: Comparing Middle Eastern Muslim refugee an immigrant adolescents. European Journal of Health Psychology.

[CR17] Emery EH, Maju M, Coursey K, Brandt C, Ko JS, Hampton K, Richards A (2022). Trauma exposures, resilience factors, and mental health outcomes in persons granted asylum in the U.S. For claims related to domestic violence and persecution by organized gangs. Journal of Immigrant and Minority Health.

[CR18] Faul F, Erdfelder E, Buchner A, Lang A-G (2009). Statistical power analyses using G*Power 3.1: Tests for correlation and regression analyses. Behavior Research Methods.

[CR19] Fazel M, Reed RV, Panter-Brick C, Stein A (2012). Mental health of displaced and refugee children resettled in high-income countries: Risk and protective factors. Lancet.

[CR20] Federal Constitutional Court [Bundesverfassungsgericht] (2020). *Jahresbericht.* https://www.bundesverfassungsgericht.de/SharedDocs/Downloads/DE/Jahresbericht/jahresbericht_2020.pdf?__blob=publicationFile&v=5

[CR21] Federal Government Expert Commission on the Framework Conditions for Integration Potential [Fachkommission Integrationsfähigkeit] (2021). *Shaping immigrations society together - Report of the federal government expert commission on the framework conditions for integration potential.* https://www.fachkommission-integrationsfaehigkeit.de/resource/blob/1786706/1827694/68198ca749da0f17c56723e3978b6b3f/englisch-data.pdf?download=1

[CR22] Federal Statistical Office [Statistisches Bundesamt] (2021). *Schutzsuchende - Ergebnisse des Ausländerzentralregisters [People seeking protection – results of the Central Register of Foreign Nationals].* https://www.destatis.de/DE/Themen/Gesellschaft-Umwelt/Bevoelkerung/Migration-Integration/Publikationen/Downloads-Migration/schutzsuchende-2010240207004.pdf?__blob=publicationFile

[CR23] Fleischmann, F. (2022). Researching religion and migration 20 years after “9/11”: Taking stock and looking ahead. *Zeitschrift für Religion, Gesellschaft und Politik, 6*. 10.1007/s41682-022-00103-6 10.1007/s41682-022-00103-6 PMC8889873 35252743

[CR24] Fleischmann F, Leszczensky L, Pink S (2019). Identity threat and identity multiplicity among minority youth: Longitudinal relations of perceived discrimination with ethnic, religious, and national identification in Germany. British Journal of Social Psychology.

[CR25] Gavranidou M, Niemiec B, Magg B, Rosner R (2008). Traumatische Erfahrungen, aktuelle Lebensbedingungen im Exil und psychische Belastung junger Flüchtlinge [Traumatic experiences, current living conditions in exile, and psychological distress of young refugees]. Kindheit Und Entwicklung.

[CR26] Greeff AP, Human B (2004). Resilience in families in which a parent has died. American Journal of Family Therapy.

[CR27] Hashemi N, Marzban M, Sebar B, Harris N (2020). Religious identity and psychological well-being among middle-eastern migrants in Australia: The mediating role of perceived social support, social connectedness, and perceived discrimination. Psychology of Religion and Spirituality.

[CR28] Hayes AF (2018). Introduction to mediation, moderation, and conditional process analysis, second edition (methodology in the social sciences).

[CR29] Holt CL, Schulz E, Williams BR, Clark EM, Wang MQ (2014). Social support as a mediator of religious involvement and physical and emotional functioning in a national sample of African-Americans. Mental Health, Religion & Culture.

[CR30] Jafari H, Kassan A, Reay G, Climie EA (2022). Resilience in refugee children and youth: A critical literature review.

[CR31] King KA, Topalian A, Vidourek RA (2020). Religiosity and adolescent major depressive episodes among 12–17-year-olds. Journal of Religion and Health.

[CR32] Kizilhan JI (2015). Religion, Kultur und Psychotherapie bei muslimischen Migranten [Religion, culture, and psychotherapy with Muslim migrants]. Psychotherapeut.

[CR33] Klein C, Albani C (2007). Religiosität und psychische Gesundheit Eine Übersicht über Befunde, Erklärungsansätze und Konsequenzen für die klinische Praxis [Religiosity and mental health. A review of findings, explanatory approaches, and implications for clinical practice]. Psychiatrische Praxis.

[CR34] Koenig HG, Al Zaben F (2021). Psychometric validation and translation of religious and spiritual measures. Journal of Religion and Health.

[CR35] Körs, A., Haddad, L., Wagner, C., & Akbaba, Y. (2022). Islamischer Religionsunterricht (IRU) in Deutschland im Spannungsfeld von Religion, Bildung, Politik und Gesellschaft [Islamic religious education (IRU) in Germany at the interplay between religion, education, politics and society]. *Zeitschrift für Religion, Gesellschaft und Politik,* 1–27. 10.1007/s41682-022-00120-5

[CR36] Kosher H, Ben-Arieh A (2017). Religion and subjective well-being among children: A comparison of six religion groups. Children and Youth Services Review.

[CR37] Krok D (2016). Sense of coherence mediates the relationship between the religious meaning system and coping styles in Polish older adults. Aging & Mental Health.

[CR38] Krok D, Zarzycka B, Telka E (2021). Religiosity, meaning-making and the fear of COVID-19 affecting well-being among late adolescents in Poland: A moderated mediation model. Journal of Religion and Health.

[CR39] Le TN, Tov W, Taylor J (2007). Religiousness and depressive symptoms in five ethnic adolescent groups. International Journal for the Psychology of Religion.

[CR40] Leszczensky, L., & Gräbs Santiago, A. (2014). *Measuring ethnic and national identity in children and adolescents (MZES Working Paper No. 155).* Mannheimer Zentrum für Europäische Sozialforschung. http://www.mzes.uni-mannheim.de/publications/wp/wp-155.pdf

[CR41] Leszczensky L, Pink S (2020). Are birds of a feather praying together? Assessing friends’ influence on Muslim youths’ religiosity in Germany. Social Psychology Quarterly.

[CR42] Liedhegener, A., Pickel, G., Odermatt, A., Yendell, A., & Jaeckel, Y. (2019). *Wie Religion «uns» trennt – und verbindet: Befunde einer Repräsentativbefragung zur gesellschaftlichen Rolle von religiösen und sozialen Identitäten in Deutschland und der Schweiz 2019 [How religion divides - and connects - "us": Findings from a representative survey on the societal role of religious and social identities in Germany and Switzerland 2019].*10.5281/ZENODO.3560792

[CR43] Lindner, K. (2022). *Gesundheitsversorgung von Asylsuchenden in den Bundesländern: Rahmenbedingungen und Reformbedarfe [Health care for asylum seekers in the federal states: Framework conditions and reform needs].* MIDEM-Policy Paper. https://ec.europa.eu/migrant-integration/system/files/2022-03/new-TUD_MIDEM_PolicyPaper_2022-1_RZ_online.pdf

[CR44] Lohaus A, Nussbeck F (2016). Fragebogen zu Ressourcen im Kindes- und Jugendalter [Questionnaire to assess resources for children and adolescents].

[CR45] Marley C, Mauki B (2019). Resilience and protective factors among refugee children post-migration to high-income countries: A systematic review. European Journal of Public Health.

[CR47] Michaels JL, Hao F, Ritenour N, Aguilar N (2022). Belongingness is a mediating factor between religious service attendance and reduced psychological distress during the COVID-19 pandemic. Journal of Religion and Health.

[CR48] Niehues, W. (2022). *Fünfte Welle der IAB-BAMF-SOEP-Befragung von Geflüchteten Entwicklung der Deutschkenntnisse, Sorgen und Lebenszufriedenheit bei Geflüchteten während des ersten Covid-19-Pandemiejahres. Ausgabe 02|2022 der Kurzanalysen des Forschungszentrums Migration, Integration und Asyl des Bundesamtes für Migration und Flüchtlinge [Fifth wave of the IAB-BAMF-SOEP survey of refugees development of german language skills, worries and life satisfaction among refugees during the first Covid-19 pandemic year. Issue 02|2022 of the brief analyses of the research center migration, integration and asylum of the Federal Office for Migration and Refugees].* https://www.bamf.de/SharedDocs/Anlagen/DE/Forschung/Kurzanalysen/kurzanalyse2-2022-iab-bamf-soep-5te-welle.pdf?__blob=publicationFile&v=8

[CR49] Ozeto, N.-F.T., & Allan, T. (2021). Investigating a relationship between perceived stress, religious coping, and religiosity in migrant Muslim women. *Journal of Muslim Mental Health, 15*(1). 10.3998/jmmh.265

[CR50] Park CL, Paloutzian RF, Park CL (2013). Religion and meaning. Handbook of the Psychology of Religion and Spirituality.

[CR51] Phalet K, Fleischmann F, Hillekens J (2018). Religious identity and acculturation of immigrant minority youth: Towards a contextual and developmental approach. European Psychologist.

[CR52] Pirutinsky S, Cherniak AD, Rosmarin DH (2020). Covid-19, mental health, and religious coping among American orthodox jews. Journal of Religion and Health.

[CR53] Powroznik N (2021). Religion in Flüchtlingsunterkünften : Sozialanthropologische Perspektiven.

[CR54] Ravens-Sieberer U, Kaman A, Erhart M, Otto C, Devine J, Löffler C, Hölling H (2021). Quality of life and mental health in children and adolescents during the first year of the COVID-19 pandemic: Results of a two-wave nationwide population-based study. European Child & Adolescent Psychiatry.

[CR55] Research group on worldviews in Germany [Forschungsgruppe Weltanschauungen in Deutschland] (2021). *Religionszugehörigkeiten 2020 [Religious affiliations 2020].* https://fowid.de/meldung/religionszugehoerigkeiten-2020

[CR56] Ronneberg CR, Miller EA, Dugan E, Porell F (2016). The protective effects of religiosity on depression: A 2-year prospective study. The Gerontologist.

[CR57] Sabatier C, Mayer B, Friedlmeier M, Lubiewska K, Trommsdorff G (2011). Religiosity, family orientation, and life satisfaction of adolescents in four countries. Journal of Cross-Cultural Psychology.

[CR58] Scharpf, F., Kaltenbach, E., Nickerson, A., & Hecker, T. (2021). A systematic review of socio-ecological factors contributing to risk and protection of the mental health of refugee children and adolescents. *Clinical Psychology Review, 83*. 10.1016/j.cpr.2020.101930 10.1016/j.cpr.2020.101930 33186775

[CR59] Schmees, P., Braig, J., Nilles, H., Kerkhoff, D., Demir, Z., Rueth, J.-E., Lohaus, A., & Eschenbeck, H. (2022). Well-being and resources of minors with refugee background in comparison to minors with migration or native background. *European Journal of Health Psychology, 29*(1), 3–14. 10.1027/2512-8442/a000099

[CR60] Schnabel L, Schieman S (2022). Religion protected mental health but constrained crisis response during crucial early days of the COVID-19 pandemic. Journal for the Scientific Study of Religion.

[CR61] Schoemann AM, Boulton AJ, Short SD (2017). Determining power and sample size for simple and complex mediation models. Social Psychological and Personality Science.

[CR62] Schweitzer R, Greenslade J, Kagee A (2007). Coping and resilience in refugees from the Sudan: A narrative account. Australian and New Zealand Journal of Psychiatry.

[CR63] Shapiro E (2022). A protective canopy: Religious and social capital as elements of a theory of religion and health. Journal of Religion and Health.

[CR64] Surzykiewicz J, Maier K, Kloubert T (2020). Spirituelle Bedürfnisse und die Lebenszufriedenheit von Flüchtlingen [Spiritual needs and the life satisfaction of refugees]. Erwachsenenbildung und Migration.

[CR65] Tan MM, Reidpath DD, Ting RS-K, Allotey P, Su TT (2022). Religiousness and quality of life among older adults of different ethnic groups in Malaysia: A five-year follow-up study. Journal of Religion and Health.

[CR66] The Federal Government Commissioner for Migration, Refugees and Integration [Die Beauftragte der Bunderegierung für Migration, & Flüchtlinge und Integration] (2019). Deutschland kann Integration: Potenziale fördern, Integration fordern, Zusammenhalt stärken [Germany can integration. Promoting potentials, demanding integration and strengthening cohesion]. https://www.bundesregierung.de/resource/blob/975292/1823792/98e849dd7baa9d2358553dbc6aa1d946/12-lagebericht-31-12-2019-download-ba-ib-data.pdf?download=1

[CR67] The KIDSCREEN Group Europe. (2006). *The KIDSCREEN questionnaires: Quality of life questionnaires for children and adolescents.* Pabst Science.

[CR68] Torralba J, Oviedo L, Canteras M (2021). Religious coping in adolescents: New evidence and relevance. Humanities and Social Sciences Communications.

[CR69] UNESCO Institute for Statistics. (2021). *Pandemic‐related disruptions to schooling and impacts on learning proficiency indicators: A focus on early grades.* https://learningportal.iiep.unesco.org/en/library/pandemic-related-disruptions-to-schooling-and-impacts-on-learning-proficiency-indicators-a

[CR70] UNICEF (2017). *Kindheit im Wartezustand: Studie zur Situation von Kindern und Jugendlichen in Flüchtlingsunterkünften in Deutschland [Childhood in wait state: Study on the situation of children and adolescents in refugee shelters in Germany].* https://www.unicef.de/download/137024/ecc6a2cfed1abe041d261b489d2ae6cf/kindheit-im-wartezustand-unicef-fluechtlingskinderstudie-2017-data.pdf

[CR71] Utsch, M. (2015). Spiritualität: Bewältigungshilfe oder ideologischer Fanatismus? Umgang mit religiös-spirituellen Ressourcen und Bedürfnissen in der Psychotherapie – Teil I [Spirituality: Coping tool or ideological fanaticism? Working with religious-spiritual resources and needs in psychotherapy - part I.]. *Psychotherapeutenjournal, 15*, 4–9. https://www.psychotherapeutenjournal.de/ptk/web.nsf/gfx/2DFA34D2680FBF42C1257F7800590226/$file/ptj_2016-1_Artikel_Utsch_Teil-I-und-II.pdf

[CR72] Utsch M, Anderssen-Reuster U, Frick E, Gross W, Murken S, Schouler-Ocak M, Stotz-Ingenlath G (2017). Empfehlungen zum Umgang mit Religiosität und Spiritualität in Psychiatrie und Psychotherapie [Recommendations for dealing with religiosity and spirituality in psychiatry and psychotherapy]. Spiritual Care.

[CR73] van Buuren S (2018). Flexible imputation of missing data.

[CR74] Vang ZM, Hou F, Elder K (2019). Perceived religious discrimination, religiosity, and life satisfaction. Journal of Happiness Studies.

[CR75] Ysseldyk R, Haslam SA, Haslam C (2013). Abide with me: Religious group identification among older adults promotes health and well-being by maintaining multiple group memberships. Aging & Mental Health.

[CR76] Zagumny MJ, Keith KD (2013). Religious Identity. The encyclopedia of cross-cultural psychology.

[CR77] Zarzycka B, Tychmanowicz A, Krok D (2020). Religious struggle and psychological well-being: The mediating role of religious support and meaning making. Religions.

[CR78] Zettl M, Akin Z, Back S, Taubner S, Goth K, Zehetmair C, Nikendei C, Bertsch K (2021). Identity development and maladaptive personality traits in young refugees and first- and second-generation migrants. Frontiers in Psychiatry.

[CR79] Zick, A., & Küpper, B. (2021). *Die geforderte Mitte: Rechtsextreme und demokratiegefährdende Einstellungen in Deutschland 2020/21 [The demanded middle: right-wing extremist and democracy-endangering attitudes in Germany 2020/21]*. Dietz. https://www.fes.de/index.php?eID=dumpFile&t=f&f=78925&token=792eddadb739a54903b934fc52256c5bbddd4428

[CR80] Zinnbauer BJ, Pargament KI, Paloutzian RF, Park CL (2005). Religiousness and spirituality. Handbook of the Psychology of Religion and Spirituality.

