# Supplementary material for: Religious Identity and its Relation to Health-Related Quality of Life and COVID-Related Stress of Refugee Children and Adolescents in Germany
Source: J Relig Health. 2023 Dec 15;63(1):765–87. doi: 10.1007/s10943-023-01966-6 (PMC10861600; doi:10.1007/s10943-023-01966-6)
Supplement: Supplementary file 1 — Supplementary file1 (DOCX 13 kb) [file 10943_2023_1966_MOESM1_ESM.docx]

**ESM 1.**

Standardized path coefficients for total, direct, and indirect effects of parallel mediation analysis with religious identity as independent variable and HRQoL as dependent variable

| Mediator | Path a | Path b | Path a x b  (indirect effect) |
| --- | --- | --- | --- |
| Ethnic identity | 0.48  (*p* < .001) | 0.12  (*p* = .068) | 0.06  Cl [-.0125; .1383] |
| Sense of coherence | 0.16  (*p* = .010) | 0.19  (*p* = .006) | 0.03  Cl [-.0006; .0771] |
| Integration into peer group | 0.27  (*p* < .001) | 0.29  (*p* < .001) | 0.08  Cl [.0259; .1448] |
| Religious practice | 0.31  (*p* < .001) | -0.13  (*p* = .023) | -0.04  Cl [-.0809; -.0061] |

*Note. N* = 246, Covariates: Religious affiliation and survey location, direct effect of religious identity on HRQoL: *b* = 0.13, *p* = .060, total effect of religious identity on HRQoL: *b* = 0.25, *p* < .001
